# Systematic screening for potential therapeutic targets in osteosarcoma through a kinome-wide CRISPR-Cas9 library

**DOI:** 10.20892/j.issn.2095-3941.2020.0162

**Published:** 2020-08-15

**Authors:** Yuanzhong Wu, Liwen Zhou, Zifeng Wang, Xin Wang, Ruhua Zhang, Lisi Zheng, Tiebang Kang

**Affiliations:** ^1^Sun Yat-sen University Cancer Center, State Key Laboratory of Oncology in South China, Collaborative Innovation Center for Cancer Medicine, Guangzhou 510060, China

**Keywords:** Osteosarcoma, kinase, CRISPR-Cas9 library, TRRAP, PKMYT1, TP53RK

## Abstract

**Objective:** Osteosarcoma is the most common primary malignant bone tumor. However, the survival of patients with osteosarcoma has remained unchanged during the past 30 years, owing to a lack of efficient therapeutic targets.

**Methods:** We constructed a kinome-targeting CRISPR-Cas9 library containing 507 kinases and 100 nontargeting controls and screened the potential kinase targets in osteosarcoma. The CRISPR screening sequencing data were analyzed with the Model-based Analysis of Genome-wide CRISPR/Cas9 Knockout (MAGeCK) Python package. The functional data were applied in the 143B cell line through lenti-CRISPR-mediated gene knockout. The clinical significance of kinases in the survival of patients with osteosarcoma was analyzed in the R2: Genomics Analysis and Visualization Platform.

**Results:** We identified 53 potential kinase targets in osteosarcoma. Among these targets, we analyzed 3 kinases, TRRAP, PKMYT1, and TP53RK, to validate their oncogenic functions in osteosarcoma. PKMYT1 and TP53RK showed higher expression in osteosarcoma than in normal bone tissue, whereas TRRAP showed no significant difference. High expression of all 3 kinases was associated with relatively poor prognosis in patients with osteosarcoma.

**Conclusions:** Our results not only offer potential therapeutic kinase targets in osteosarcoma but also provide a paradigm for functional genetic screening by using a CRISPR-Cas9 library, including target design, library construction, screening workflow, data analysis, and functional validation. This method may also be useful in potentially accelerating drug discovery for other cancer types.

## Introduction

Osteosarcoma (OS) is a bone cancer that most commonly affects children, adolescents during the adolescent growth spurt, and young adults. The incidence of osteosarcoma is approximately 1 to 3 cases annually per million worldwide^1^. Osteosarcoma primarily arises in children and adolescents, and a second peak in incidence is found in people over 50 years of age^2^. Before the 1970s, osteosarcoma was rarely curable, even with ablative surgery. Since then, the combination of surgical resection and chemotherapy has resulted in a 5-year overall survival rate of ~70% in patients with localized disease^3^. However, approximately 15%–20% of patients have clinically detectable metastases at the time of diagnosis. More than 85% of the metastatic disease occurs in the lungs, and the bone is the second most frequent site of distant disease. Unfortunately, the survival rate for patients with metastatic or relapsed osteosarcoma has remained virtually unchanged over the past 30 years, with an overall 5-year survival rate of only 20%^4,5^. The current standard treatment for osteosarcoma is neoadjuvant chemotherapy followed by surgical resection, which results in a 3-year event-free survival ranging from 50% to 75% and a 5-year survival ranging from 60% to 85%^6^. To substantially increase the survival rates, multiple clinical trials of novel agents including target drugs have been initiated. Unfortunately, no tyrosine kinase inhibitors, such as sorafenib, pazopanib, and imatinib, have shown more promising outcomes than traditional therapies, although many receptor tyrosine kinases, including platelet derived growth factor, HER2 (also known as ERBB2), VEGF, IGF1, and MET, are overexpressed in osteosarcoma^7–9^. Blockade of the PD-1/PD-L1 interaction has not provided convincing evidence of a benefit for patients with osteosarcoma^10–12^. In addition, the Aurora inhibitors VX-680 and ZM447439 display toxicity in osteosarcoma cell lines and induce hyperploidy and apoptosis; however, their clinical efficiencies must be validated in clinical trials^13^. A comprehensive understanding of the molecular mechanisms underlying osteosarcoma and the discovery of new therapeutic targets is urgently needed.

shRNA libraries have long been the dominant method used to systematically identify the functional genes in cancers, although this approach has several shortcomings, including low efficiency and off-target effects^14^. In recent years, the RNA-guided endonuclease Cas9, a component of the type II clustered, regularly interspaced, short palindromic repeats (CRISPR) system of bacterial host defense, was developed as a powerful tool for genome editing^15,16^. Ectopic expression of Cas9 and a single guide RNA (sgRNA) is sufficient to produce a DNA double-strand break at a specific gene locus and induce indel formation and gene knockout. Several groups have recently demonstrated that the CRISPR-Cas9 library is an easy and efficient system for performing high-throughput loss-of-function screening^17–19^. Through whole genome CRISPR-Cas9 knockout library screening, Shalem et al*.*^17^ have found that knockout of NF1, MED12, NF2, CUL3, TADA1, and TADA2B confers resistance to the BRAF inhibitor vemurafenib (PLX) in melanoma cells. In addition, Tim Wang and colleagues, through genome-scale screening, have reported that knockout of cyclin-dependent kinase 6 (CDK6) confers resistance to the DNA topoisomerase II poison etoposide^18^. In another report, Zachary Steinhart and colleagues, through genome-wide CRISPR screening, have revealed that the Wnt–FZD5 signaling circuit is a druggable vulnerability of RNF43-mutant pancreatic tumors^20^. Yamauchi et al.^21^ have also screened and identified leukemia-specific dependence on a pre-mRNA metabolic pathway regulated by DCPS and have validated that a DCPS inhibitor, RG3039, has selective toxicity against AML but not normal blood cells. All these studies have demonstrated that the CRISPR-Cas9 library screening platform has great potential for therapeutic target identification and can aid in cancer drug discovery.

Despite the great success achieved with CRISPR-Cas9 library screening, the screening process remains a major challenge because most ready-to-use libraries are genome-scale, containing tens of thousands sgRNAs in single pool^22^. Maintaining the plasmids, producing virus and representing sgRNAs in cells is difficult; because of these restrictions, genome-scale libraries usually contain only 4–6 sgRNAs per gene^19,17,23^. Moreover, the knockout efficiency of every sgRNA is not ensured, thus potentially producing false negative results^24^. Therefore, using small libraries targeting only specific gene families with more sgRNAs per gene is desirable to increase the accuracy, sensitivity, and specificity^25^. Kinases are intimately involved in cancer growth, proliferation, and survival, and they are frequently mutated in the genome^26,27^. Kinases have emerged as the largest class of cancer drug targets, thus prompting us to systematically investigate potential therapeutic kinase targets in osteosarcoma. In this report, we generated a kinome-wide CRISPR-Cas9 library containing 507 kinases with 10 sgRNAs per gene. Through this focused library, we screened for kinase essentiality by using the 143B, MG63, and U2OS/MTX300 cell lines. We found that 53 kinases are essential for the survival of these osteosarcoma cell lines, with an FDR < 5%. These genes clustered in the functional categories of cell cycle, MAPK pathway, and transcription processes. Among the 53 genes, PLK1, integrin-linked protein kinase (ILK), transformation/transcription domain-associated protein (TRRAP), PKMYT1, and TP53RK were hits in all 3 cell lines, and PLK1 and ILK have been reported to be oncogenes in osteosarcoma. We further validated that TRRAP, PKMYT1, and TP53RK are required for osteosarcoma cell proliferation and colony formation. In conclusion, we revealed the overall landscape of the kinase dependency in osteosarcoma cell lines through unbiased CRISPR-Cas9 screening, which may shed light on osteosarcoma drug discovery in the future.

## Materials and methods

### Kinome-wide CRISPR-Cas9 library construction

The sgRNA sequences were designed according to reference^18^. Briefly, 10 candidate sgRNAs per gene were designed according to the following criteria: (1) targeting constitutive exons in different isoforms, (2) closest downstream of the start codon, and (3) GC content between 20% and 80%. One hundred random nontargeting control sgRNAs were also generated and filtered by specificity. This kinome-wide library contained 5070 sgRNAs designed for 507 kinases, plus 100 nontargeting control sgRNAs, for a total of 5170 sgRNAs.

The oligonucleotide sgRNA sequences were synthesized with a CustomArray 12K chip (CustomArray Inc.). The sequences of oligonucleotides with flanking adaptors were GTGGAAAGGACGAAACACCGXXXXXXXXXXXXXXXXXXXXGTTTTAGAGCTAGAAATAGC, and the oligonucleotide pool was amplified by PCR with the following primers with homologous arms to the lenti-CRISPR V2 plasmid: 

F: TAACTTGAAAGTATTTCGATTTCTTGGCTTTATATATCTTGTGGAAAGGACGAAACACCG,

R: ACTTTTTCAAGTTGATAACGGACTAGCCTTATTTTAACTTGCTATTTCTAGCTCTAAAAC

The PCR products were separated by electrophoresis and purified with a TIANGEN DNA purification kit according to the manufacturer’s directions. The lenti-CRISPR V2 plasmid was digested by Esp3I and separated by electrophoresis, and then the upper linearized plasmid band was extracted. Gibson assembly was used to insert the oligonucleotides into the lenti-CRISPR V2 plasmid according to the manufacturer’s directions. The reaction system is shown in **Table 1**.

The assembly products were electroporated into electrocompetent cells according to the manufacturer’s directions. Four rounds of electroporation were repeated to obtain a sufficient number of clones, the transformed cells were plated onto large LB agar plates, and a series of dilutions were performed to calculate the clone number to ensure at least 300-fold coverage of the number of kinome sgRNAs. Colonies were harvested from LB agar plates, and maxi preps were performed with an OMEGA plasmid maxi kit.

### Screening procedure

To produce the lentivirus, ten 10 cm dishes of HEK293T cells were transfected with the library. For each dish, 12 μg of kinome-wide library plasmid, 8 μg of pSPAX2, and 4 μg of pMD2G were mixed in 1 mL of opti-MEM, and then 100 μL of PEI was added, gently mixed, incubated for 10 min, and added to HEK293T cells. Six hours later, the medium was changed, and the culture was retained. Forty-eight hours later, the medium was collected and filtered through a 0.45 μm PVDF membrane. The multiplicity of infection (MOI) of the virus for 143B, MG63, and U2OS/MTX300 cells was calculated individually according to reference^17^. The cells were infected at 0.3 MOI, and 10,000,000 cells were plated into five 10 cm dishes for each cell line. Forty-eight hours later, the cells were passaged with puromycin (0.5 μg/mL) selection for another 3 days. These cells were denoted generation 0 (G0). At this stage, we separated the cells into 2 parts: half was extracted for genomic DNA, and half was kept for culturing. Both parts ensured at least 500-fold coverage of the sgRNA library to represent the whole library, meaning that at least 2,500,000 cells were harvested or passaged. After proliferation for 16 generations, the genomic DNA was extracted and denoted G16. The sgRNAs were amplified from G0 and G16 with the following primers:

F: TCTTGTGGAAAGGACGAAACACCG

R: CCTAGCTAGCGAATTCAAAAAAGCAC

The resulting PCR products were applied to next-generation sequencing. Using the Illumina platform and PE150 sequencing, at least 3 G clean data were obtained for each sample. The data were analyzed with the MAGeCK package.

### MAGeCK analysis

The Python package Model-based Analysis of Genome-wide CRISPR/Cas9 Knockout (MAGeCK)^28^ was used to identify essential genes from the kinome-wide CRISPR-Cas9 library knockout screens. MAGeCK performs well in controlling the false discovery rate (FDR) and has high sensitivity. MAGeCK is able to perform both positive and negative selection screens simultaneously.

### Cell lines and reagents

The 143B, MG63, and HEK293T cell lines were cultured according to the instructions from the ATCC. U2OS/MTX300 cells were cultured as described previously^29^. Antibodies to TRRAP (GTX129507-S, GeneTex), PKMYT1 (4282T, Cell Signaling Technology), and TP53RK (26818-1-AP, Proteintech); Cisplatin (T1564, Topscience); and lenti-CRISPR V2, pSPAX2, and pMD2.G (Addgene) were used.

### Gene knockout by CRISPR-Cas9

The sgRNA sequence was synthesized and annealed, then ligated into lenti-CRISPR V2, which had been linearized by Esp3I. The plasmids were then transformed into Stbl3 *E. coli*, and the sequences were confirmed by Sanger sequencing. The primers used are shown in **Table 2**.

### Western blot

The 143B cell line was infected with the indicated lentivirus, and 7 days later, the cells were lysed in RIPA lysis buffer (50 mM Tris–HCl, 150 mM NaCl, 5 mM EDTA, and 0.5% Nonidet P-40 and a protease and phosphatase inhibitor cocktail (Calbiochem)). Proteins were separated by sodium dodecyl sulphate-polyacrylamide gel electrophoresis and transferred to 0.45 μm PVDF membranes (Millipore). The immunoblots were processed according to standard procedures by using primary antibodies directed against TRRAP, PKMYT1, and TP53RK.

### Proliferation assay

Cell proliferation was determined with MTT assays. Briefly, 143B cells were infected with the indicated lentivirus, and 7 days later, the cells were plated on 96-well plates with 1,000 cells per well. After 1, 2, 3, 4, or 5 days of proliferation, MTT was added to each well under sterile conditions (at a final concentration of 5 mg/mL), and the plates were incubated for 4 h at 37 °C. Untransformed MTT was removed by aspiration, and formazan crystals were dissolved in dimethyl sulfoxide (100 μL/well). The plate was shaken for 10 min until the crystals dissolved. The absorbance was measured at 490 nm with an Infinite F50 microplate reader (Tecan).

### Colony formation assays

Colony formation assays were performed as described previously. The 143B cell line was infected with the indicated lentivirus, and 5 days later, the cells were plated in triplicate at 300 cells per well in 6-well plates and then cultured for 8 days. The cell clones were washed with PBS, fixed in methanol, and dyed with 0.1% crystal violet, and the colonies containing more than 50 cells were counted.

### Apoptosis assays

The 143B cell line was infected with the indicated lentivirus, and 7 days later, cells were plated into 6-well plates in triplicate; 24 h later, the cells were treated with cisplatin or were left untreated at a 5 μg/mL concentration for 12 h to induce apoptosis. Cells were then collected with trypsin without EDTA, washed with PBS, subjected to annexin V-FITC and propidium iodide staining according to the manufacturer’s recommendations (KeyGen Biotech), and analyzed by flow cytometry.

### Survival analysis

All analyses of the osteosarcoma patient data were conducted in the R2: Genomics Analysis and Visualization Platform (http://r2.amc.nl), and the resulting figures and *P* values were downloaded.

## Results

### Design, establishment, and quality control of the kinome-wide CRISPR-Cas9 library

**Figure 1:**
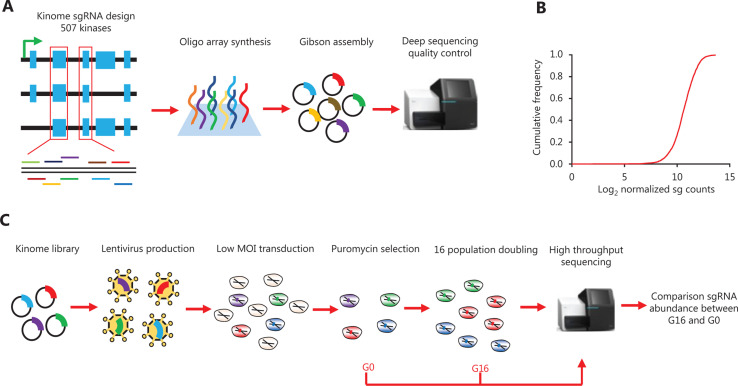
Design, establishment, and quality control of the kinome-wide CRISPR-Cas9 library.

To establish a kinome-wide CRISPR-Cas9 library, we designed sgRNA sequences for the 507 kinases in the human genome. The sgRNA sequences were based on the work of Tim et al.^18^ and were designed against the constitutive exons shared by different isoforms of the genes, then filtered for potential off-target sequences, on the basis of similarities to the genome. Although the efficiency and specificity will probably vary for different sequences, false-positive and false-negative sgRNAs in screens can still be mitigated by designing redundant sgRNAs for each gene, a process requiring multiple distinct sgRNAs targeting the same gene to display the same phenotype. We used 10 sgRNAs for each gene, a number greater than that in typical genome-scale libraries, which usually contain only 4–6 sgRNAs per gene^19,23,25^. One hundred nontargeting controls were also included in this library, an aspect critical to evaluating the noise in the screening. We simultaneously synthesized sgRNA oligonucleotides containing left and right arms by using a high-throughput chip array. We then amplified these sequences with left and right arm primers by using PCR. Through Gibson assembly, we successfully cloned the sgRNAs into the lenti-CRISPR V2 backbone (**Figure 1A**). To assess the effective representation of this kinome-wide library, we conducted amplification and high-throughput sequencing of the sgRNAs, and we were able to detect nearly all the sgRNAs that we had designed. Furthermore, uniformity across the constructs showed only a sevenfold increase between the 10th and 90th percentiles (**Figure 1B**).

### Workflow of the CRISPR-Cas9 screening in osteosarcoma cell lines

To screen for kinase dependency in osteosarcoma, we established a screening workflow as follows (**Figure 1C**). First, we transfected the sgRNA library with virus package helper plasmids into HEK293T cells. After 48 h, we collected and filtered the virus and calculated the MOI. Second, we transduced the 143B, MG63, and U2OS/MTX300 cells at a low MOI (~0.2), and 48 h later, we selected the cells with puromycin for 72 h. We then obtained the sgRNA-transduced cells, which we denoted generation 0 (G0). In this step, we separated the cells into 2 parts; half was extracted for the genomic DNA, and half was kept for culturing. Both parts ensured at least 500-fold coverage of the sgRNA library to represent the whole library, meaning that at least 2,500,000 cells were harvested or passaged. After 16 generations, we extracted the genomic DNA and denoted it G16. Finally, we amplified the sgRNAs from G0 and G16 and applied next-generation sequencing.

### Statistics of the screening results

**Figure 2:**
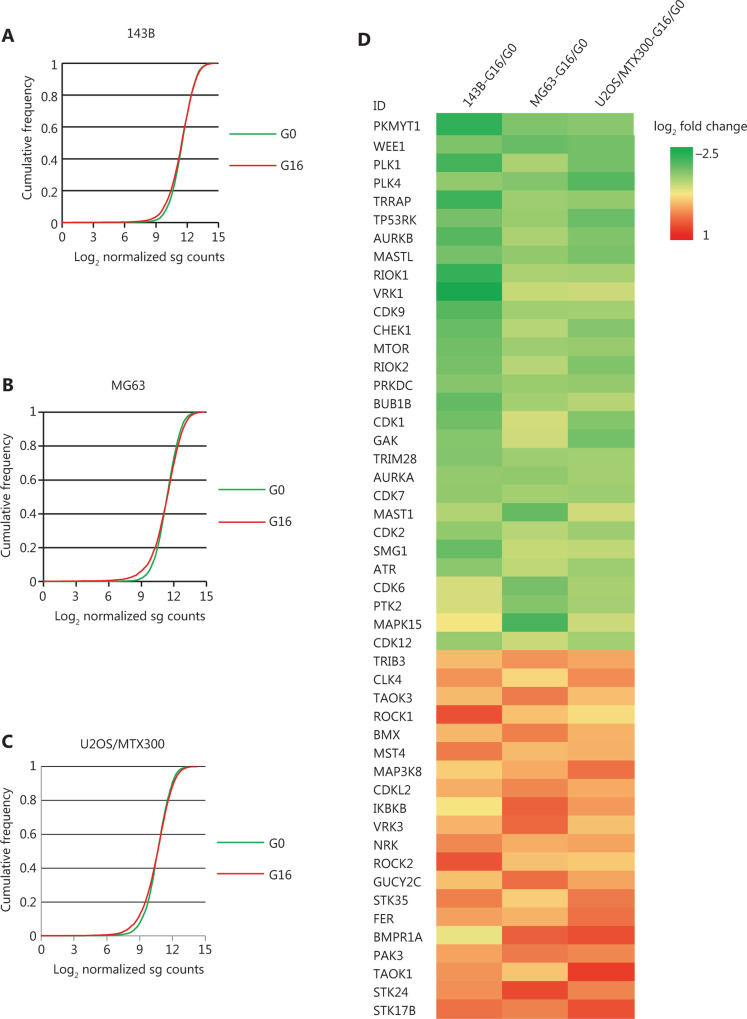
Statistics of the CRISPR screening results.

**Figure 3:**
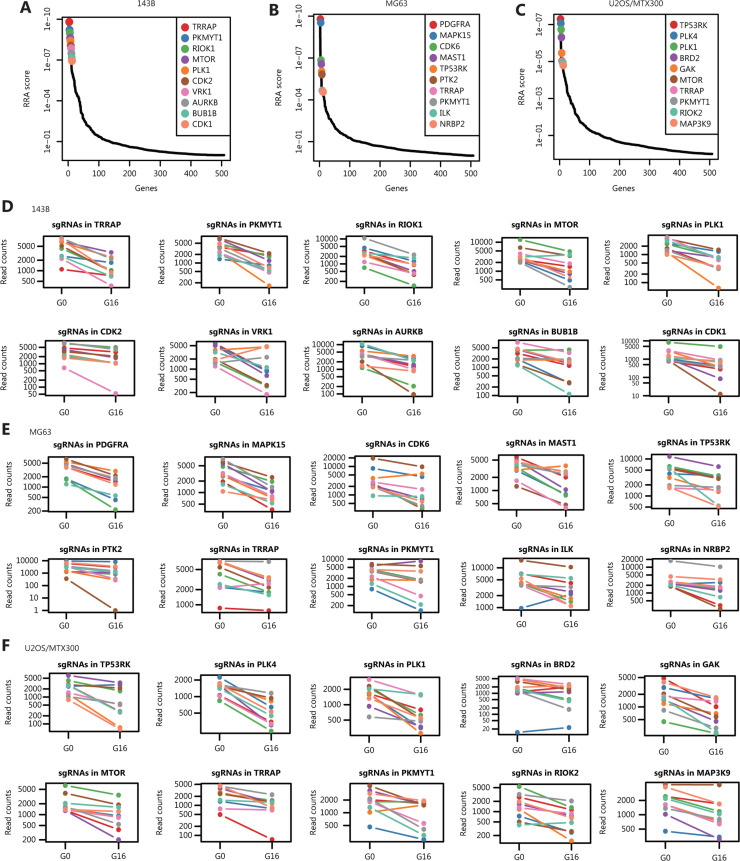
Representation of the top 10 significant genes.

To analyze the essentiality of the kinases, we compared the G16 and G0 compositions of each sgRNA by using the MAGeCK algorithm, which can assess the statistical significance of sgRNA ranking with the binomial model before identifying the targets^28^. **Figure 2A–2C** show the cumulative frequencies of sgRNAs for G16 and G0 in the indicated cell lines, and the shift in the G16 curve represents the depletion of a subset of sgRNAs. The heatmap in **Figure 2D** shows the fold change in sgRNA abundance of G16 relative to G0 in the 143B, MG63, and U2OS/MTX300 cells, and the full results of the changes are shown in **Supplementary Table S1**, which includes the statistics of the screening results in these 3 cell lines. Using a 5% FDR as the cut-off, we identified 41, 17, and 24 essential genes for 143B, MG63, and U2OS/MTX300 cells, respectively (**Table 3**). The top 10 significant genes whose knockout induced proliferation inhibition in 143B, MG63, and U2OS/MTX300 cells are shown in **Table 4**. **Figure 3A–3C** show the RRA scores from MAGeCK analysis in the indicated cell lines. The changes in individual sgRNA counts between G16 and G0 in the sequencing data are presented in **Figure 3D–3F**.

### Analyses of the pathway enrichment and protein-protein interaction network of the essential genes in osteosarcoma

**Figure 4:**
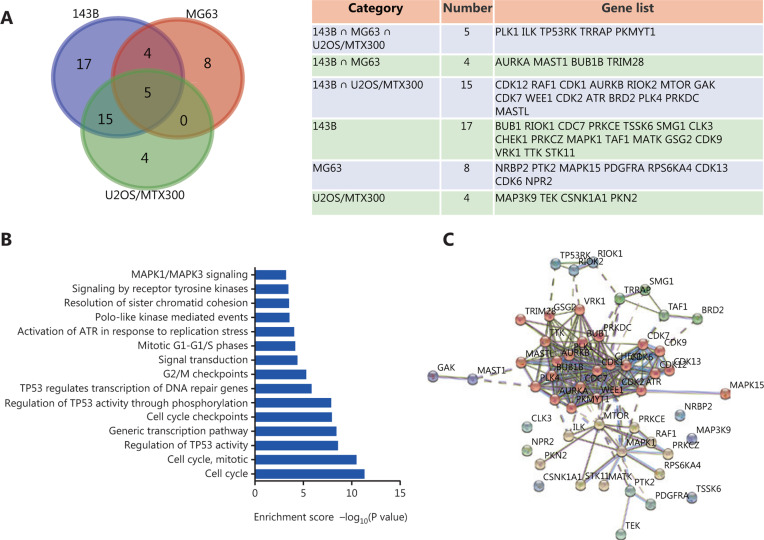
Pathway enrichment and protein-protein interaction network of the essential genes in osteosarcoma.

To understand the mechanisms underlying the identified essential kinases, we analyzed the composition of essential genes. As shown in **Figure 4A** (left), a Venn diagram of the significant genes in 143B, MG63, and U2OS/MTX300 cells showed 53 essential genes in these cell lines, among which 5 genes, PLK1, ILK, TRRAP, PKMYT1, and TP53RK, were identified in all cell lines (right). We then analyzed the pathway enrichment for these essential kinases. As shown in **Figure 4B**, these genes were primarily enriched in the cell cycle and TP53-related pathways. Protein-protein interaction analysis revealed 224 edges in this network; the nodes were mainly clustered into 3 groups, the largest of which was cell cycle-related kinases; the second was MTOR and MAPK1-related signal pathways; and the third was mRNA transcription and decay-related molecules, including TRRAP, TAF1, SMG1, and BRD2 (**Figure 4C**).

### Functional validation and clinical relevance of TRRAP, PKMYT1, and TP53RK in osteosarcoma

**Figure 5:**
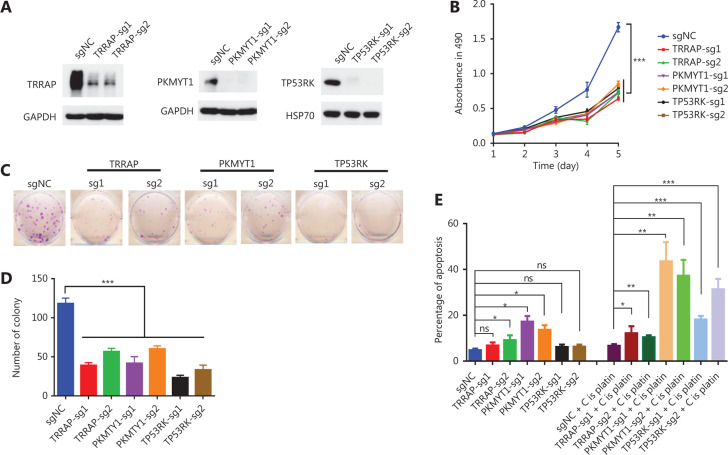
Functional validation of TRRAP, PKMYT1, and TP53RK in osteosarcoma.

**Figure 6:**
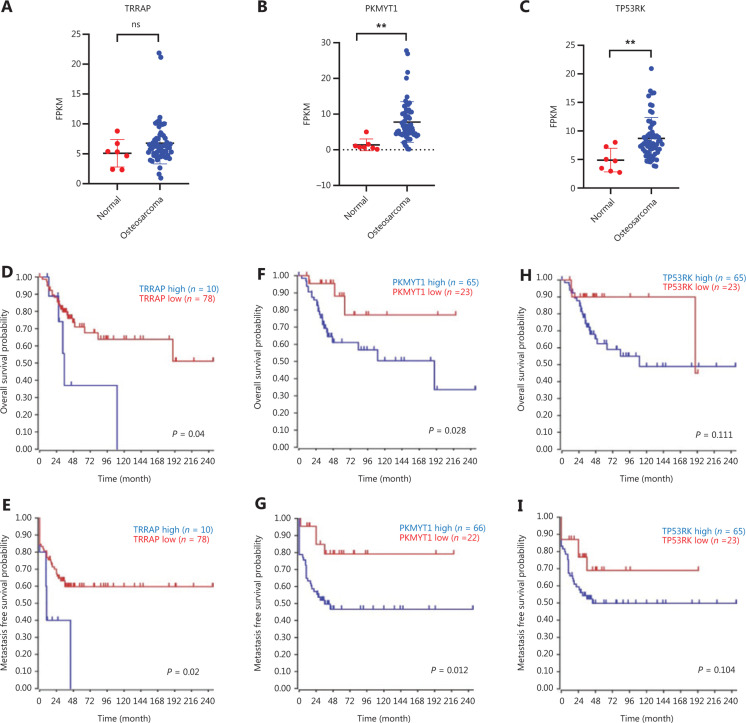
TRRAP, PKMYT1, and TP53RK gene expression levels and clinical relevance in patients with osteosarcoma.

Two hits from our screen, PLK1 and ILK, have been reported to be oncogenes in osteosarcoma. Consequently, we decided to validate whether the 3 other hits from our screening, TRRAP, PKMYT1, and TP53RK, were truly essential in osteosarcoma cell lines, given that their functions in osteosarcoma remain unknown^30,31^. First, we knocked out TP53RK, TRRAP, and PKMYT1 by using 2 different sgRNAs for each gene, and we validated the knockout efficiency through Western blot (**Figure 5A**). We then assessed the biological functions of the knockout cells, as shown in **Figure 5B–5D**, and observed markedly diminished colony formation ability and proliferation rates in the knockout cells. Moreover, apoptosis induced by cisplatin, the most widely used chemotherapy drug for osteosarcoma, was dramatically elevated in these knockout cells, although stable knockout of PKMYT1 alone also induced apoptosis in 143B cells. We further investigated the expression levels of these 3 kinases in osteosarcoma and normal bone tissues. Combining 2 RNA-Seq datasets recently published by our group and another group^32,33^, we found that PKMYT1 and TP53RK were overexpressed in osteosarcoma, whereas TRRAP showed no difference between osteosarcoma and normal bone tissue (**Figure 6A–6C**). Finally, we evaluated the clinical relevance in the survival of patients with osteosarcoma by using R2: Genomics Analysis and Visualization Platform. Only 88 patients had survival information in the Mixed Osteosarcoma (Mesenchymal)-Kuijjer-127 dataset. Patients with high levels of either TRRAP or PKMYT1 had lower rates of both overall survival and metastasis-free survival than those with low levels of each gene (**Figure 6D–6G**). Although the survival curves did not significantly differ between patients with low and high TP53RK levels, the patients with high levels of TP53RK tended to have lower rates of both overall survival and metastasis-free survival (**Figure 6H, 6I**). We speculate that a greater sample size might have yielded significant results for both survival rates.

## Discussion

The lack of effective therapeutic targets has resulted in a standstill in the survival of patients with osteosarcoma over the past 30 years. Recently, CRISPR-Cas9 screens in cancer survival, metastasis, immune marker expression, viral infection, and other biological processes have yielded impressive results^34–39^. These studies have demonstrated that CRISPR-Cas9 libraries are a powerful discovery platform for the rapid understanding of cellular processes, thus prompting us to identify new potential therapeutic targets in osteosarcoma.

Protein kinases are one of the largest protein families in the human genome^26^. Recent advances in the understanding of cancer cells have revealed the crucial roles of kinases in carcinogenesis and metastasis. Constitutive overexpression of some kinases may be associated with oncogenesis^26^. High-throughput sequencing has also indicated the causality between mutations or fusions of some kinases with cancers, the most famous examples of which are L858R and T790M EGFR mutations in lung cancer and BCR-ABL fusion genes in chronic myeloid leukemia^40,41^. Here, we successfully constructed a kinome-wide CRISPR-Cas9 library containing 507 kinases and 100 nontargeting controls. Through systematic screening, we identified 53 kinases as potential targets in osteosarcoma at 5% FDR levels. Reactome pathway analysis showed that these genes are functionally enriched primarily in the cell cycle and TP53-related pathways. Protein-protein interaction analysis revealed that these genes clustered into 3 major groups. As shown in **Figure 4C**, the cell cycle-related kinases accounted for the largest group in the map, and knockout of CDK1, CDK2, WEE1, and other critical molecules could reasonably be expected to disrupt the cell cycle and induce cell death. The second largest group was mTOR, RAF1, and MAPK1-related kinases, which are involved in cancer cell metabolism and signal transduction. Activated mTOR contributes to osteosarcoma transformation and indicates a poor prognosis; it functions through downstream effectors such as eIF4, 4EBP1, and S6K1^42^. RAF1-MAPK1 is one of the best-established oncogenic pathways in cancers, including osteosarcoma^43^. The third group was transcription-related genes, including TAF1, TRRAP, BRD2, and SMG1. The first 3 of these genes are associated with gene transcription, whereas SMG1 is responsible for nonsense-mediated decay of mRNAs containing premature stop codons, through phosphorylation of UPF1/RENT1^44^. BRD2 is an atypical protein kinase that has mitogen-activated nuclear kinase activity and is involved in signal transduction; its kinase domain is located between the 2 bromodomains^45^. Our screens indicated that BRD2 may be a potential target for osteosarcoma, which deserves further investigation in the future.

Five kinases, PLK1, ILK, TRRAP, PKMYT1, and TP53RK, were essential in the 143B, MG63, and U2OS/MTX300 cell lines according to our screen. PLK1 and ILK have been reported to be oncogenes in osteosarcoma^31,30^. PLK1 is highly expressed during mitosis, and elevated levels are found in many cancers, including osteosarcoma, whereas depletion of PLK1 in osteosarcoma cells dramatically inhibits cell proliferation and induces apoptosis. Furthermore, GSK461364, a potent and selective ATP-competitive PLK1 inhibitor, exerts a cytotoxic effect by inducing apoptosis and senescence in osteosarcoma cell lines^30^. ILK is a receptor-proximal protein kinase regulating integrin-mediated signal transduction. overexpression of ILK is significantly correlated with distant metastasis and poor survival in patients with osteosarcoma, whereas downregulation of ILK significantly increases apoptosis and decreases angiogenesis and invasiveness of osteosarcoma cells^31^. On the basis of this knowledge of PLK1 and ILK, we speculate that the other 3 shared kinases, TRRAP, PKMYT1, and TP53RK, may also be potential targets for osteosarcoma, as shown in **Figure 5 and 6**.

TRRAP belongs to the phosphatidylinositol 3-kinase-related kinase family, which includes ATM, DNA-PKcs, ATR, and TRRAP. TRRAP was originally identified as an essential cofactor for the c-Myc and E2F oncoproteins^46^. Further studies have shown that it is a component of the histone acetyltransferase complex, and it assists in the recruitment of the histone acetyltransferase complex to chromatin during gene transcription^47,48^. TRRAP has also been shown to be involved in cell cycle progression, chromatin remodeling, and embryonic development^49^. Overexpression of TRRAP has been reported to promote stem cell characteristics in gliomas^50^. However, its roles in osteosarcoma have not been reported. Our data demonstrated its oncogenic functions in osteosarcoma (**Figure 5 and 6**), although the underlying mechanisms warrant further investigation.

PKMYT1 is well known for its functions in cell cycle control. It acts as a negative regulator of entry into mitosis, predominantly by phosphorylating CDK1 on threonine 14. Data have shown that PKMYT1 plays an essential oncogenic role in colorectal cancer and hepatocellular carcinoma, and it may serve as a good therapeutic target for cancer treatment^51^. On the basis of **Figure 5E**, we speculate that PKMYT1 inhibitors may be promising for the treatment of osteosarcoma, whether as single inhibitors or in combination with cisplatin. The development of PKMYT1 inhibitors is ongoing, and structural analysis and computer-aided design have led to the identification of several novel inhibitors active in the submicromolar range^52^, thus providing a basis for targeting PKMT1 in multiple cancers, including osteosarcoma.

TP53RK (p53-related protein kinase) is an atypical protein kinase first identified in an interleukin-2–activated cytotoxic T-cell subtraction library. Purified TP53RK can be activated by AKT phosphorylation at serine 250^53^. Elevated expression of TP53RK is observed in colorectal cancer and is associated with poor prognosis^54^, and its functions are both p53-dependent and independent^55^. Here, we showed that knockout of TP53RK dramatically decreased the proliferation and colony formation ability in 143B cells, although knockout of TP53RK itself did not affect its apoptosis but dramatically sensitized the cells to cisplatin treatment. The immunomodulatory drug Pom binds TP53RK and inhibits its kinase activity^55^; therefore, Pom may also be a potential drug for osteosarcoma.

## Conclusions

In summary, the kinome-wide CRISPR-Cas9 library provides a powerful tool for genetic screening in osteosarcoma and may also be useful to potentially accelerate drug discovery for other cancer types. Our results not only identify potential therapeutic kinase targets for osteosarcoma but also provide a paradigm for functional CRISPR-Cas9 library genetic screening, including target design, library construction, screening workflow, data analysis, and functional validation.

## Figures and Tables

**Table 1:** Reaction system for Gibson assembly

Component	Amount
Gibson Assembly Master Mix	10 μL
Digested lenti-CRISPR V2	200 ng
Purified oligonucleotide PCR product	20 ng
Ultra-pure water	To 20 mL

**Table 2:** Primer sequences for sgRNA clone

Primers	Sequence
TRRAP-sg1-F	CACCGGCGGCAGTCACCAGGTGCAG
TRRAP-sg1-R	AAACCTGCACCTGGTGACTGCCGCC
TRRAP-sg2-F	CACCGGAAGCAAGGAGAAAAGGACA
TRRAP-sg2-R	AAACTGTCCTTTTCTCCTTGCTTCC
PKMYT1-sg1-F	CACCGGGGCCATGGCTCCTACGGAG
PKMYT1-sg1-R	AAACCTCCGTAGGAGCCATGGCCCC
PKMYT1-sg2-F	CACCGAACATGGAGCTGCCCCACGG
PKMYT1-sg2-R	AAACCCGTGGGGCAGCTCCATGTTC
TP53RK-sg1-F	CACCGAGAGCTACTACGCCGGCCGA
TP53RK-sg1-R	AAACTCGGCCGGCGTAGTAGCTCTC
TP53RK-sg2-F	CACCGGCTGGTGAAGCAGGGTGCCG
TP53RK-sg2-R	AAACCGGCACCCTGCTTCACCAGCC

**Table 3:** Statistics of screening hits in the 3 cell lines at different FDR levels

Comparison	Selection	FDR 1%	FDR 5%	FDR 25%
143B-G16/G0	Negative	36	41	59
MG63-G16/G0	Negative	11	17	40
U2OS/MTX300-G16/G0	Negative	15	24	50

**Table 4:** The top 10 significant genes whose knockout induced cell death in 143B, MG63, and U2OS-MTX300 cells

143B				MG63				U2OS/MTX300			
ID	neg|score	neg|*P*	neg|fdr	ID	neg|score	neg|*P*	neg|fdr	ID	neg|score	neg|*P*	neg|fdr
TRRAP	1.76E-10	9.75E-06	2.48E-04	PDGFRA	1.82E-10	9.75E-06	8.25E-04	TP53RK	5.44E-08	9.75E-06	9.90E-04
PKMYT1	6.80E-10	9.75E-06	2.48E-04	MAPK15	3.14E-10	9.75E-06	8.25E-04	PLK4	9.25E-08	9.75E-06	9.90E-04
RIOK1	1.14E-09	9.75E-06	2.48E-04	CDK6	1.58E-07	9.75E-06	8.25E-04	PLK1	1.98E-07	9.75E-06	9.90E-04
MTOR	2.66E-09	9.75E-06	2.48E-04	MAST1	2.96E-07	9.75E-06	8.25E-04	BRD2	5.22E-07	9.75E-06	9.90E-04
PLK1	4.32E-09	9.75E-06	2.48E-04	TP53RK	1.01E-06	9.75E-06	8.25E-04	GAK	3.68E-06	9.75E-06	9.90E-04
CDK2	9.89E-09	9.75E-06	2.48E-04	PTK2	1.64E-06	9.75E-06	8.25E-04	MTOR	1.01E-05	2.92E-05	1.65E-03
VRK1	1.58E-08	9.75E-06	2.48E-04	TRRAP	2.37E-05	1.66E-04	9.41E-03	TRRAP	1.10E-05	2.92E-05	1.65E-03
AURKB	3.73E-08	9.75E-06	2.48E-04	PKMYT1	2.53E-05	1.85E-04	9.41E-03	PKMYT1	1.19E-05	2.92E-05	1.65E-03
BUB1B	7.57E-08	9.75E-06	2.48E-04	ILK	2.69E-05	1.85E-04	9.41E-03	RIOK2	1.22E-05	2.92E-05	1.65E-03
CDK1	1.28E-07	9.75E-06	2.48E-04	NRBP2	2.92E-05	1.85E-04	9.41E-03	MAP3K9	1.71E-05	6.82E-05	3.47E-03
